# Using a Web-Based Quiz Game as a Tool to Summarize Essential Content in Medical School Classes: Retrospective Comparative Study

**DOI:** 10.2196/22992

**Published:** 2021-04-29

**Authors:** Varah Yuenyongviwat, Jongdee Bvonpanttarananon

**Affiliations:** 1 Department of Orthopedics Faculty of Medicine Prince of Songkla University Hatyai Thailand

**Keywords:** medical education, medical students, computer games, gaming, web-based, interface, perception, retrospective

## Abstract

**Background:**

Kahoot! is a web-based technology quiz game in which teachers can design their own quizzes via provided game templates. The advantages of these games are their attractive interfaces, which contain stimulating music, moving pictures, and colorful, animated shapes to maintain students’ attentiveness while they perform the quizzes.

**Objective:**

The aim of this study was to evaluate the use of Kahoot! compared with a traditional teaching approach as a tool to summarize the essential content of a medical school class in the aspects of final examination scores and the perception of students regarding aspects of their learning environment and of process management.

**Methods:**

This study used an interrupted time series design, and retrospective data were collected from 85 medical students. Of these 85 students, 43 completed a Kahoot! quiz, while 42 students completed a paper quiz. All students attended a lecture on the topic of bone and joint infection and participated in a short case discussion. Students from both groups received the same content and study material, with the exception that at the end of the lesson, students in the Kahoot! group completed a quiz summarizing the essential content from the lecture, whereas the other group received a paper quiz with the same questions and the teacher provided an explanation after the students had finished. The students’ satisfaction was evaluated after the class, and their final examination was held 2 weeks after the class.

**Results:**

The mean final examination score in the Kahoot! group was 62.84 (SD 8.79), compared to 60.81 (SD 9.25) in the control group (*P*=.30). The students’ satisfaction with the class environment, learning process management, and teacher were not significantly different between the 2 groups (all *P*>.05).

**Conclusions:**

In this study, it was found that using Kahoot! as a tool to summarize the essential content in medical school classes involving a lecture and case discussion did not affect the students’ final examination scores or their satisfaction with the class environment, learning process management, or teacher.

## Introduction

Game-based learning is a teaching method that integrates games into the learning process. Game-based learning uses “game mechanics,” in which tools or applications are used to produce motivation, interactivity, and rewards [1]. Kahoot is a web-based technology quiz game that enables teachers to design their own quizzes in provided game templates. The advantages of this game are its attractive interface, which contains stimulating music, moving pictures and colorful, animated shapes, which can maintain students’ attentiveness while they complete the quiz [2,3].

One study reported that the majority of students using Kahoot! reported sentiments such as “I have fun and I learn,” and that it reinforced what they had learned in class [4]. However, in health care education, there are limited studies that evaluate using Kahoot! in the classroom as a tool to summarize essential content, as compared with traditional teaching approaches in which students complete the quiz on paper and the teacher summarizes the essential content after the quiz. Therefore, the aim of this study was to evaluate the results of using Kahoot! in the aspects of final examination scores and the perceptions of students regarding the learning environment and process management compared with traditional teaching approaches.

## Methods

The design of this study involved an interrupted time series and retrospective data collection. Data from fifth-year medical students who attended a bone and joint infection class in the Orthopedic Department of the Faculty of Medicine, Prince of Songkla University, between April 2017 and March 2019 were retrieved from the undergraduate medical education unit database. We compared students who used Kahoot! in the classroom as a tool to summarize the essential content of the class between April 2018 and March 2019 with students who attended class between April 2017 and March 2018 and who completed a paper quiz with the teacher summarizing the essential content after the quiz as the control group. This study was approved by the Ethics Committee and Institutional Review Board of the Faculty of Medicine, Prince of Songkla University. Consent was waived by the ethics committee. The faculty gave permission for the extraction of this information from the database.

All students attended a lecture on the topic of bone and joint infection and a short case discussion, with each class containing 10-12 students. All students in the Kahoot! group and control group received the same content and study material, with the exception of the end of the lesson, wherein students in the Kahoot! group completed a quiz in Kahoot! to summarize the essential content of the lecture while the other group completed a paper quiz; both quizzes contained the same questions. The quiz for both groups was presented on the screen in front of the classroom. In the Kahoot! group, all students completed the quiz via their mobile phone. Each quiz consisted of a process and time limit; after answering each question, the students progressed to the next question. The rules of the game were that the student who provided the most correct answers was the winner; during the quiz, after answering each question, the total score and the score leader’s name were shown. The quiz consisted of 10 questions; each question had four answer choices with a single correct answer, and the teacher provided a short explanation after each question in the Kahoot! group. In the control group, the quiz was completed by the students on paper, and the teacher gave an explanation after students had finished the entire quiz.

The students’ satisfaction with the class environment, learning process management, and teacher was evaluated by a numeric rating scale in which 0 represented “least satisfied” and 4 indicated “most satisfied.” This assessment was conducted through a web-based evaluation program. The evaluation process was performed after the class, and the results for each student were blinded to the identity of the student to prevent information bias from the student, while the teacher gave feedback. All the students wrote a final examination 2 weeks following the class, with the same examination questions in both groups.

The analyses were conducted using R version 3.1.0 software (R Foundation for Statistical Computing). Student grade point average (GPA), satisfaction in each domain, and examination score were evaluated with the Student *t*-test. The Pearson chi-square test was used for a comparison of gender between the groups. The sample size estimation was performed based on previous student examination scores (mean 63.7, SD 8). For each group, 25 students were required to detect a 10% difference in the examination scores with a significance level set to *P*=.05 and a power set to 0.8.

## Results

A total of 85 students were included in this study. Of these 85 students, 43 played Kahoot! and 42 students used a traditional method. There were no significant differences in gender between the 2 groups (Kahoot! group: 26 female and 17 male students; control group: 29 female and 13 male students, *P*=.41). The GPAs of the students were also not significantly different between the 2 groups (Kahoot! group: 3.32, SD 0.3; control group: 3.21, SD 0.26; *P*=.07).

The mean examination score in the Kahoot! group was 62.84 (SD 8.79), compared to 60.81 (SD 9.25) in the control group (*P*=.30). The students’ satisfaction with the class environment, learning process management, and teacher were not significantly different between the 2 groups (Table 1).

**Table 1:** Mean student satisfaction scores for the 2 groups (N=85). Scores ranged from 0-4, with 0 indicating low satisfaction and 4 indicating high satisfaction.

Variable			Kahoot! group (n=43), mean (SD)	Control group (n=42), mean (SD)		*P* value
**Promoting a good learning environment**						
	Interaction between teachers and students	3.9 (0.38)		3.88 (0.33)	.81	
**Learning process management**						
	Learning process management that emphasizes student participation	3.9 (0.3)		3.83 (0.38)	.38	
	Using media and learning resources	3.75 (0.44)		3.86 (0.35)	.23	
	Organizing the learning process so that the learned material can be applied	3.93 (0.27)		3.88 (0.33)	.51	
**Evaluation**						
	Evaluation during teaching	3.88 (0.33)		3.93 (0.26)	.42	
**Teacher**						
	Teaching and personality	3.93 (0.27)		3.88 (0.33)	.68	
	Encouraging learners to demonstrate proper behavior, including respecting students	3.98 (0.16)		3.88 (0.33)	.11	

## Discussion

### Principal Findings

In our study, we found that the final examination scores for students who used Kahoot! in the classroom as a tool to summarize essential content were slightly higher compared with those of students who learned the same material through traditional teaching approaches and completed the quiz on paper; however, this difference did not reach statistical significance. It should be noted that our results are in contradiction with those in previous reports. In a study of business course students by Bawa [5], it was found that students in classes using Kahoot! had significantly better scores on their final examinations than students in a control group. Nevertheless, there is one study that supports our results. A study of the use of Kahoot! in an introductory-level animal science course by Harrison [6] showed that students in the Kahoot! group did not have significantly higher examination scores compared with students in the control group.

In this study, we found that student satisfaction with the class environment, learning process management, and teacher were not significantly different between the Kahoot! group and control group. This result was the same as that in a previous study of high school students learning Chinese as a foreign language [7]. The results of that study showed that use of Kahoot! by students had no significant effect on student motivation. In other research on the use of Kahoot! compared with traditional methods in an Earth Science class [8], it was also found that there were no significant differences in the students’ overall learning motivation or in any of the motivation variables, such as motivation, value, expectation, and emotional experience, between the 2 groups.

### Limitations

This study had a number of limitations. First, this study had a limited number of participants; therefore, this study was likely underpowered due to the lower than expected differences in outcomes. Second, the satisfaction evaluated in this study was overall satisfaction with a class that consisted of a lecture, case discussion, and either a Kahoot! quiz or paper quiz. In our study, Kahoot! was only used at the end of the class. We hypothesized that students would prefer Kahoot! to a paper quiz; however, the impact of Kahoot! may not have been large enough to change the overall satisfaction score of the class.

### Conclusion

This study found that using Kahoot! as a tool to summarize the essential content in medical school classes that involved both a lecture and case discussion did not affect students’ final examination scores. Additionally, it did not affect student satisfaction with the class environment, learning process management, or teacher.

### Data Availability

The data sets generated during this study are available from the corresponding author upon reasonable request.

## Abbreviations

GPA: grade point average
